# Impact of ethnicity and extreme prematurity on infant pulmonary function

**DOI:** 10.1002/ppul.22882

**Published:** 2013-09-30

**Authors:** Ah-Fong Hoo, Amit Gupta, Sooky Lum, Kate L Costeloe, Angela Huertas-Ceballos, Neil Marlow, Janet Stocks

**Affiliations:** 1 Portex Respiratory Unit, UCL Institute of Child HealthLondon, WC1N 1EH, UK; 2 Paediatric Respiratory Medicine Unit, Great Ormond Street Hospital for Children NHS Foundation TrustLondon, WC1N 3JH, UK; 3 Neonatal Unit, John Radcliffe HospitalOxford, OX3 9DU, UK; 4 Neonatal Unit, Homerton University Hospital NHS Foundation TrustLondon, E9 6SR, UK; 5 Academic Unit of Paediatrics, Barts and the London School of Medicine and Dentistry, Queen Mary University of LondonLondon, E1 2AT, UK; 6 Neonatal Office, University College London Hospital NHS Foundation TrustLondon, NW1 2PG, UK; 7 Academic Neonatology, UCL Elizabeth Garrett Anderson Institute for Women's Health, University College LondonLondon, WC1E 6AU, UK

**Keywords:** extremely preterm, lung function tests, ethnic background, plethysmography, raised volume technique

## Abstract

The impact of birth before 27 completed weeks of gestation on infant pulmonary function (PF) was explored in a multi-ethnic population in comparison to more mature preterm controls (PTC) and healthy fullterm infants. Plethysmographic lung volume (FRC_pleth_) and forced expired volume (FEV_0.5_) were obtained at ∼12 months post-term age in 52 extremely preterm (EP) infants (median [range] gestational age [GA]: 26 [23–27] weeks; 40% White mothers; 79% with BPD), 41 PTC (GA:35 [30–36] weeks; 37% White mothers) and 95 fullterm infants (GA:40 [37–42] weeks; 86% White mothers). Using reference equations based on identical equipment and techniques, results were expressed as *z*-scores to adjust for age, sex and body size. FEV_0.5_ was significantly lower in EP infants when compared with PTC (mean difference [95% CI]: −1.02[−1.60; −0.44] *z*-scores, *P* < 0.001), as was forced vital capacity (FVC) but there were no significant differences in FRC_pleth_ or FEV_0.5_/FVC ratio. FEV_0.5_, FVC, and FEV_0.5_/FVC were significantly lower in both preterm groups when compared with fullterm controls. On multivariable analyses of the combined preterm dataset: FEV_0.5_ at ∼1 year was 0.11 [0.05; 0.17] *z*-scores higher/week GA, and 1.28 (0.49; 2.08) *z*-scores lower in EP infants with prior BPD. Among non-white preterm infants, FEV_0.5_ was 0.70 (0.17; 1.24) *z*-scores lower, with similar reductions in FVC, such that there were no ethnic differences in FEV_0.5_/FVC. Similar ethnic differences were observed among fullterm infants. These results confirm the negative impact of preterm birth on subsequent lung development, especially following a diagnosis of BPD, and emphasize the importance of taking ethnic background into account when interpreting results during infancy as in older subjects.

## INTRODUCTION

Preterm birth is one of the most important factors influencing an infant's subsequent health and survival, with long term adverse effects on pulmonary function (PF) throughout childhood and into early adulthood.^1^–^4^ Advanced obstetric and neonatal care has resulted in increased survival of extremely preterm (EP) infants but the impact of extreme prematurity on lung development during infancy has yet to be determined. While a significant improvement in survival was noted in the 2006 EPICure cohort of EP infants when compared to that recruited in 1995, the pattern of major neonatal morbidity and the proportion of survivors affected were unchanged, indicating an important increase in the number of preterm survivors at risk of later health problems.^5^

It is now recognized that much of the burden of adult pulmonary disease has its origins in early life^6^ with “tracking” of PF throughout the life-course, that is, those with lower PF in the early years tending to retain this position thereafter.^7^ However, the true impact of prematurity on subsequent lung development can only be determined if other important determinants, including somatic growth and ethnic origin, are taken into account.^8^,^9^ When compared with women of other ethnic backgrounds, Black women are 3–4 times more likely to have a very early preterm birth,^10^,^11^ and ethnic differences in neonatal respiratory morbidity have been reported.^12^

The current observational study explored the impact of extreme prematurity (EP) on PF in a multi-ethnic urban UK population by comparing results from infants born EP (EP: defined as birth up to and including 26 weeks and 6 days gestation, hereafter referred to as <27 weeks) at ∼1-year post-term age (where “term” is defined as 40 weeks post-menstrual age), with those from more mature “healthy” preterm control (PTC) infants of similar age and ethnic background, after adjustment for age, sex and body size. Results were also compared with those from healthy fullterm infants studied during a similar time period using identical equipment and methods. We hypothesized that, after adjusting for potential confounders, PF at 1 year of age would be significantly associated with gestational age.

## MATERIALS AND METHODS

### Subjects

Infants born <27 weeks gestation were recruited between September 2006 and March 2008 from the Neonatal Unit at the Homerton University or University College Hospitals, London. A contemporaneous control group (born ≥27 but <37 weeks gestation) comprising preterm infants who required minimal ventilatory support (no invasive ventilation, <6 hr nasal continuous positive airway pressure (CPAP) and/or ≤48 hr supplemental inspired oxygen (O_2_) following delivery) and without any neonatal respiratory problems was also recruited. Results from both groups were compared with those from fullterm healthy infants who were recruited as controls for on-going clinical and epidemiological research studies (2002–2011).^13^,^14^ Approval for this study was granted by UCL Institute of Child Health/Great Ormond Street Hospital Research Ethics Committee (REC#05/Q0508/141) and written parental consent was obtained for all infants.

### Infant Pulmonary Function Tests

All pulmonary function assessments were measured by the same investigators using identical protocols and equipment at either the Homerton Hospital or UCL Institute of Child Health. Measurements were undertaken when infants were clinically well and free of symptoms, with an interval of ≥2 weeks since any respiratory symptoms. Infants were examined clinically and weighed prior to sedation with oral chloral hydrate (60–100 mg/kg). Crown-heel length was measured using a calibrated stadiometer. Weight and length at time of test were expressed as *z*- (i.e., standard deviation) scores to adjust for sex and post-term age.^15^ Pulmonary function tests (PFTs) were undertaken between 7 and 15 months post-term age during behaviourally determined “quiet” sleep. The Jaeger Masterscreen BabyBody System (v.4.65; CareFusion, Yorba Linda, CA) was used to measure plethysmographic functional residual capacity (FRC_pleth_)^16^,^17^ followed by forced expiratory maneuvers from an inflation pressure of 30 cmH_2_0 using the raised volume technique.^18^ PFT results were expressed as *z*-scores to adjust for post-term age, height and sex, using reference equations derived from healthy fullterm infants, many of whom were studied during the same time period, by the same team, using identical methods and equipment.^16^,^19^

### Data Management and Sample Size

Following quality control, PFT results were electronically exported to a research database (Re-Base™ software, Re-Base Ltd., London, UK), which also contained all relevant demographic and clinical details. Data analyses were undertaken using IBM SPSS Statistics (v20, Armonk, New York): comparisons between the two preterm study groups were analyzed using independent sample *t-*tests with 95% confidence intervals (CI) for continuous data and chi-square or Fisher exact tests where appropriate for categorical outcomes. Comparisons between the two preterm groups and fullterm infants were undertaken using ANOVA. Multivariable regression analyses were performed to estimate the association of prematurity, BPD and ethnicity with pulmonary function. Taking into account two primary outcomes (FEV_0.5_ and FRC_pleth_), a sample size of 43 infants per group would allow detection of differences equivalent to 0.5 *z*-scores with 90% power at the 5% significance level.^20^

## RESULTS

**Fig 1:**
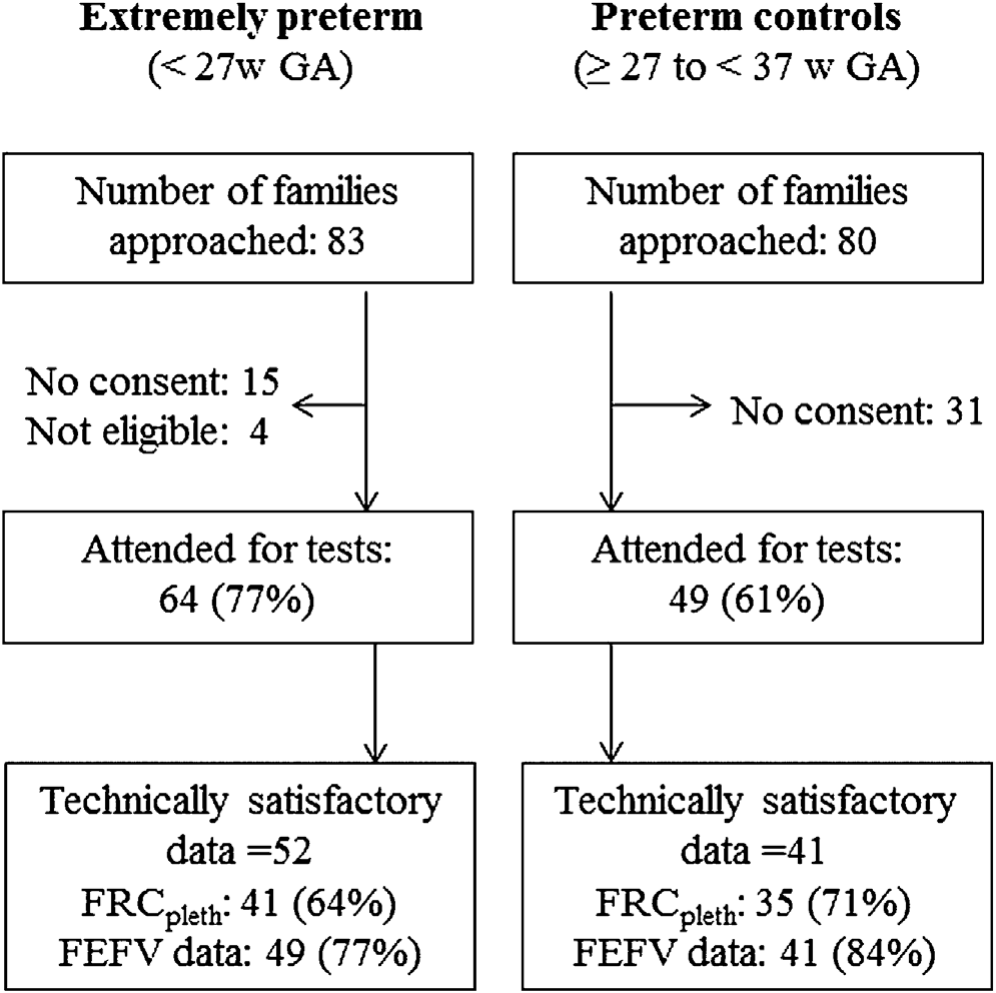
Flow diagram showing success rates in relation to recruitment and achievement of technically acceptable infant pulmonary function outcomes. Footnote: w, weeks; GA, gestational age; FEFV, forced expiratory flow-volume data from the raised volume technique.

In total, PFTs were attempted in 113 preterm infants representing 77% of EP and 61% PTCs whose families were approached (Fig. 1). Technically acceptable datasets were available from 93 infants, with similar success rates (∼80%) for both groups (Fig. 1).

Results were available from 52 EP infants (GA: range 23.4–26.9 weeks) and 41 PTC (GA: range 30.0–36.4 weeks; Table 1). There were no differences in sex distribution, birth-weight *z*-score or exposure to pre- or post-natal tobacco smoke between groups. Significantly more (24% vs. 8%) PTC than EP infants were born small-for-gestational age (SGA: birthweight <10th percentile for gestation), with twice as many girls than boys being born SGA in both these groups (data not shown). Cotinine analysis of infant urine [median (range): 0.9 (0.1–20.4) ng/ml] or maternal saliva [median (range): 0.2 (0.1–2.2) ng/ml] at time of test in reported non-smoking mothers were compatible with no active smoking (i.e., <50 ng/ml in urine and <12 ng/ml in saliva).^21^ Ninety percent (43/52) of those born EP received antenatal steroids, with 94% receiving postnatal surfactant therapy. Duration of supplemental oxygen for the EP group during the neonatal period ranged from 1 to 96 (median: 28.6) days, while that of intermittent positive pressure ventilation, with and without nasal CPAP, ranged from 13 to 143 (median: 64.5) days and 1–116 (median: 25.5) days, respectively. Seventy-nine percent (41/52) of EP subjects were still receiving supplemental oxygen at 36 weeks post-menstrual age and were thus classified as having had bronchopulmonary dysplasia (BPD) (46% with moderate BPD; 33% with severe BPD).^22^ Significantly more EP infants had been prescribed bronchodilators and/or had experienced lower respiratory tract illness (LRI) since discharge from the neonatal unit when compared with PTC infants (Table 1). Twelve percent EP infants required hospital admission due to LRI prior to testing; none of the PTCs had been hospitalized.

**Table:** Comparison of Background Characteristics in Preterm Infants

	EP (n = 52)	PTC (n = 41)	Δ (95% CI), EP–PTC
Boys (%)	25 (48%)	26 (63%)	−15% (−34%; 5%)
Gestational age (weeks)	25.6 (1.0)	34.7 (1.3)	−9.1 (−9.6; −8.6)***
Birth weight (kg)	0.80 (0.1)	2.21 (0.5)	−1.4 (−1.6; −1.2)***
Birth weight *z*-score^1^	−0.3 (0.7)	−0.4 (1.1)	0.1 (−0.3; 0.5)
Birthweight <10th percentile^1^ (%)	4/52 (8%)	10/41 (24%)	−17% (−32%; −2%)*
Maternal smoking, antenatally	5 (10%)	3 (7%)	2% (−11%; 14%)
Maternal smoking, postnatally	5 (10%)	10 (24%)	−15% (−31%; 0%)
Maternal antenatal steroids	43 (90%)^2^	20 (50%)^3^	40% (21%; 56%)**
PROM >24 hr	20 (51%)^4^	12 (31%)^4^	21% (−1%; 40%)
Prior respiratory morbidity			
(a) Bronchodilator ever	19/42 (45%)	5/38 (13%)	32% (12%; 49%)**
(b) LRI post discharge	12/42 (29%)	2/38 (5%)	23% (7%; 39%)**

Data presented as n (%) or mean (SD).

EP, extremely preterm; PTC, preterm control; PROM, prolonged rupture of membranes; LRI, lower respiratory illness.

Δ mean difference between groups.

1 Calculated using the UK-WHO algorithm.^15^

2 n = 48.

3 n = 40.

4 n = 39.

* *P* ≤ 0.05.

** *P* ≤ 0.01.

*** *P* ≤ 0.001.

Table 2 compares demographics at time of test and pulmonary function results between the two groups of preterm infants, with results from healthy fullterm infants included for comparison. A similar percentage of EP and PTC infants were born to White European mothers (∼40%), whereas twice as many fullterm infants were White. At the time of test, the EP group was slightly older than the PTCs. After correction for age and sex, weight, length and body mass index were similar at time of test between the two preterm groups but were lower than that observed in fullterm infants (Table 2).

**Table:** Demographics at Time of Test and Pulmonary Function Results

		EP versus PTC		
	Term controls (n = 95)	(n = 52)	(n = 41)	Δ (95% CI) EP–PTC
Age (weeks)^+^	48.7 (14.2)	58.2 (11.8)	48.0 (6.8)	10.3 (6.4; 14.1)***
Weight *z*-score^≠^	0.5 (1.0)	−0.20 (1.3)	−0.003 (1.0)	−0.2 (−0.7; 0.3)
Length z-score^≠^	0.8 (1.1)	0.11 (1.4)	0.59 (1.2)	−0.5 (−1.0; 0.1)
BMI *z*-score	0.03 (0.9)	−0.35 (1.1)	−0.45 (0.9)	0.1 (−0.3; 0.5)
Ethnicity				
White mothers	82 (86%)	21 (40%)	15 (37%)	4% (−16%; 23%)
Black mothers^1^	10 (10%)	22 (42%)	14 (34%)	8% (−12%; 27%)
Other^2^	3 (3%)	9 (17%)	12 (29%)	−12% (−29%; 5%)
Pulmonary function results				
6FRC_pleth_, *z*-score^3^	−0.13 (1.1)	−0.06 (1.8)	−0.52 (1.0)	0.5 (−0.2; 1.1)
7FEV_0.5_ *z*-score^4^	0.17 (0.9)	−1.62 (1.4)	−0.61 (1.4)	−**1.0** (−**1.6;**−0.4)***
7FVC *z*-score^4^	0.15 (1.0)	−0.92 (1.1)	−0.17 (1.1)	−**0.8** (−**1.2;** −0.3)***
7FEF_25–75_ *z*-score^4^	0.06 (1.0)	−1.8 (1.7)	−0.74 (1.4)	−**1.1** (−**1.7;** −0.5)***
7FEV_0.5_/FVC *z*-score^5^	−0.12 (0.8)	−0.89 (0.8)	−0.62 (0.9)	−0.3 (−0.6; 0.1)

Data presented as mean (SD) or n (%).

Δ (95% CI) in **bold font** denotes significant differences in body size and pulmonary function results between the two preterm groups. Differences with respect to the fullterm group were assessed using ANOVA and are summarized in text.

EP, extremely preterm; PTC, preterm controls; BMI, body mass index.

Δ, mean difference between groups; +, age at time of test corrected for gestation; ≠, calculated using the UK-WHO algorithm.^15^

*** *P* ≤ 0.001 for *t*-tests between EP and PTC groups.

1 Including Afro-Caribbean and African mothers.

2 Includes infants born to mothers of mixed ethnic origins, Asian or Chinese mothers.

3 Calculated according to Nguyen et al.^16^

4 Calculated according to Lum et al.^19^

5 Calculated according to Jones et al.^23^

6 n = 41 for EP, 35 for PTC and 74 for term controls for FRC_pleth_.

7 n = 49 for EP, 41 for PTC and 89 for term controls for forced expiratory volumes and flows.

**Fig 2:**
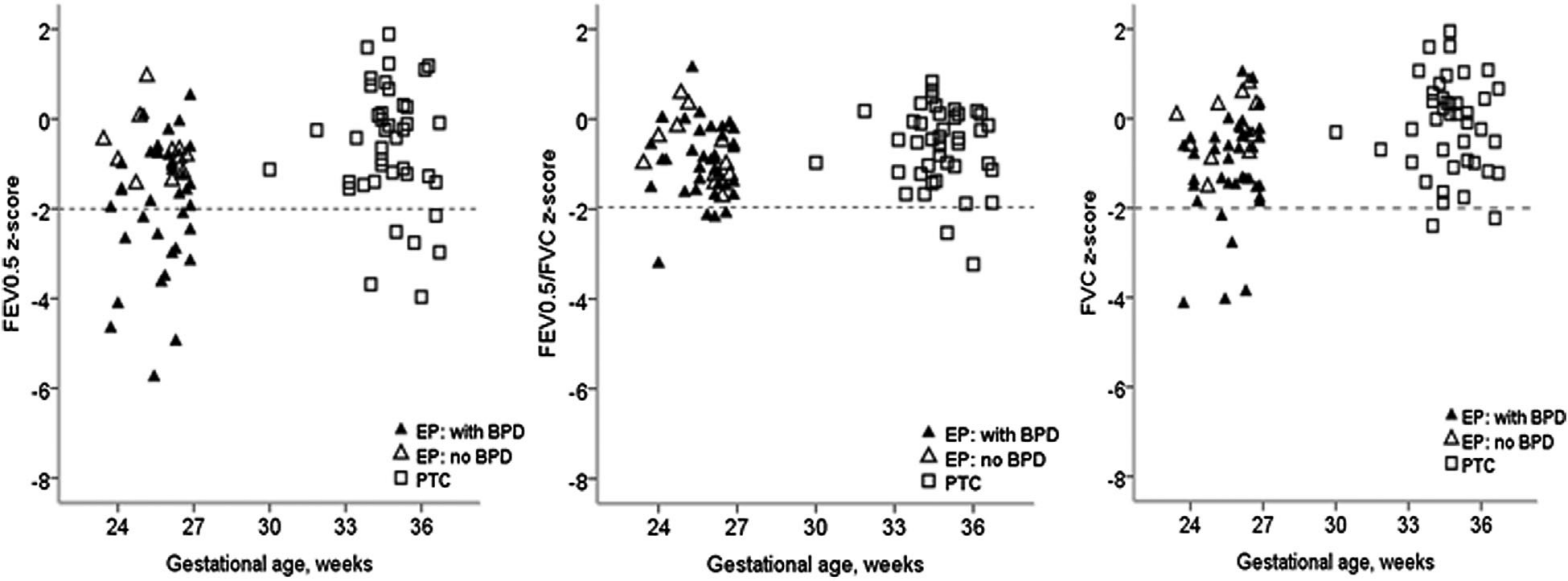
The influence of bronchopulmonary dysplasia and gestational age on pulmonary function at ∼1 year of age in infants born preterm. Footnote: EP, extremely preterm infants (n = 52), PTC, preterm control infants (n = 41), BPD, bronchopulmonary dysplasia. Dashed lines indicate the lower limit of normality (−1.96 *z*-scores) in healthy fullterm infants.^19^,^23^ Symbols: Solid and open triangles represent EP infants with and without BPD, respectively; open squares denote preterm controls.

After adjusting for body size, age and sex,^16^ there was no significant differences in FRC_pleth_ between the preterm groups, although values in PTC were 0.5 (−0.2; 1.1) *z*-scores lower than in EP infants. By contrast, forced expiratory volumes (FEV_0.5_ and FVC) and flows (FEF_25–75_) were significantly lower in EP children when compared to PTCs (all *P* < 0.001; Table 2 and Fig. 2). The FEV_0.5_/FVC *z*-score^23^ was somewhat, but not significantly, lower in those born EP than in PTC (*P* = 0.15). Neither background demographics nor PFT results were influenced by the center from which infants had been recruited (data not shown). When compared with results from the 95 fullterm healthy infants of similar age, whose mean (SD) *z*-scores for all pulmonary function outcomes were close to the expected values of 0(1),^24^ FEV_0.5_, FEF_25–75_ and FEV_0.5_/FVC were significantly lower in both groups of preterm infants (Table 2).

Univariable analyses were undertaken within the EP group to explore the associations between PFT outcomes and potential explanatory variables. After adjustment for body size, age and sex by expressing results as *z*-scores, there were no additional significant determinants of FRC_pleth_. By contrast, gestational age, administration of surfactant, mechanical and/or CPAP ventilation, BPD, birth-weight, male sex, having a White mother, and maternal smoking postnatally were all independently and significantly associated with FEV_0.5_, FEF and FVC at ∼1 year of age.

**Fig 3:**
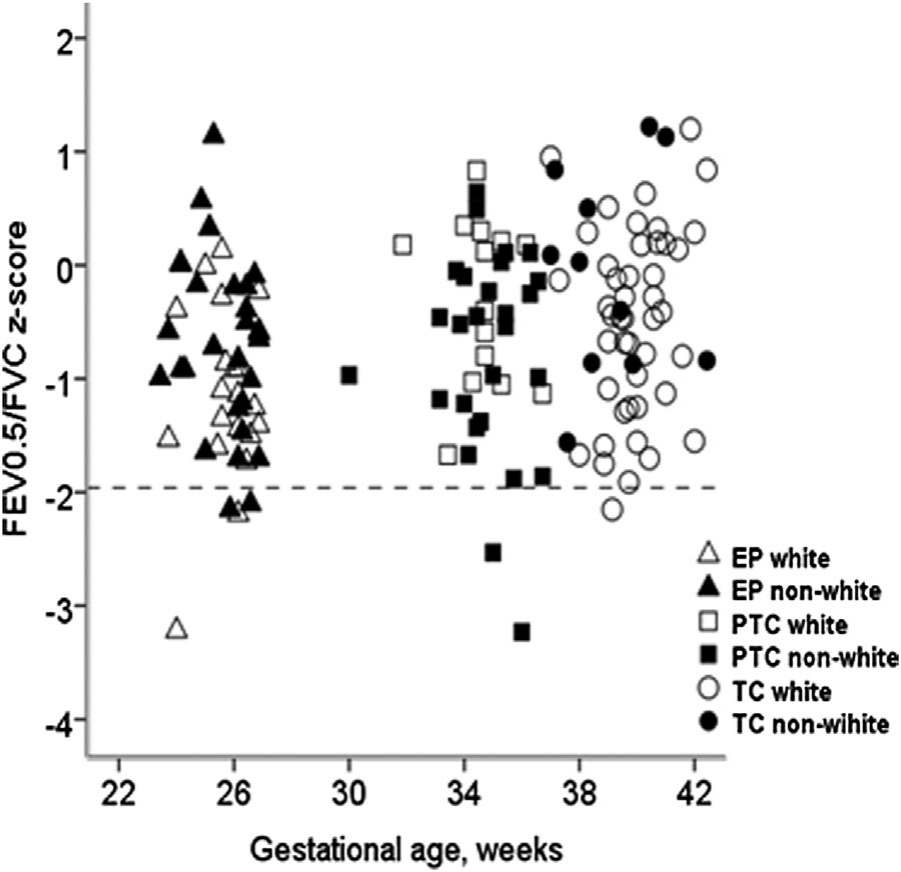
The association between FEV_0.5_/FVC *z*-scores and gestational age according to ethnicity. Footnote: Symbols: triangles represent extremely preterm infants, squares: preterm controls and circles: fullterm controls. Open and solid symbols denote those born to white and non-white mothers, respectively.

When these explanatory variables were included in multivariable analyses of the entire preterm dataset, only gestational age *or* BPD, having a White mother and being male remained significantly associated with FEV_0.5_ and FVC. On multivariable analyses, each additional week of GA was associated with increment of 0.10 (0.04; 0.16) *z*-score for FEV_0.5_ and 0.08 (0.03; 0.13) *z*-score for FVC at ∼1 year of age; similar results being observed for FEF_25–75_ (data not shown). Prior diagnosis of BPD was associated with a reduction in FEV_0.5_ and FVC *z*-scores by −1.7 [−2,4; −1.1], and −1.2 [−1.7; −0.7], respectively (Fig. 2), although GA was no longer significant once BPD was included in this model. On multivariable analysis, FEV_0.5_ was 0.70 (0.17; 1.24) *z*-score lower among non-white preterm infants, with similar reductions in FVC, such that there was no difference in FEV_0.5_/FVC. These ethnic differences were of similar magnitude when analysis was restricted to the 36 “Black,” rather than all the “non-white” preterm infants. Amongst preterm infants, boys had significantly higher values of FEV_0.5_ and FVC than girls (0.98 [95% CI: 0.40; 1.57] and 0.66 [0.18; 1.13] *z*-scores, respectively). The FEV_0.5_/FVC ratio was not influenced by sex, ethnicity or prior BPD, the only significant association being with GA; increasing by 0.05 (0.03; 0.07) *z*-score for each additional week of gestation (Fig. 3).

When expressing PF data from the fullterm healthy infants as *z*-scores (Table 2), results were, as expected, independent of sex, age and body size. Despite the relatively low numbers, FEV_0.5_, FVC, and FRC_pleth_ were all significantly lower in “non-white” infants by 0.74 (95% CI: 0.20; 1.28, *P* = 0.008), 0.84 (0.24; 1.43, *P* = 0.006) and 0.52 (0.06; 0.98, *P* = 0.029) *z*-scores, respectively. As in the preterm groups, the FEV_0.5_/FVC ratio was independent of ethnicity (Fig. 3).

## DISCUSSION

This study demonstrates clear evidence of diminished lung function at ∼1-year of age in infants born EP when compared either with contemporaneous PTCs or with healthy fullterm infants, after adjusting for important determinants including age, sex, body size, and ethnicity. Lung function was significantly associated with gestational age, and was significantly diminished even in PTCs without neonatal lung disease when compared to healthy fullterm infants. Among the EP children, this association appeared to be largely mediated through the concomitant diagnosis of BPD which remains so prevalent in such immature infants.^5^,^25^ After adjustment for BPD or gestational age, age, and body size, both FEV_0.5_ and FVC (but not their ratio) were significantly lower among non-white infants than in their white peers.

The strengths of this study include the fact that although infants were tested on two sites, all were assessed by the same team using identical methods and equipment, with equipment-specific reference data being used to adjust for age, sex and body size at time of test to avoid any bias in interpretation.^16^,^19^ We have previously demonstrated that use of appropriate reference equations is essential for interpretation of infant forced expiratory flow-volume (FEFV) and FRC_pleth_ data,^16^,^19^ collected using the infant Jaeger Masterscreen system, in order to avoid mis-diagnosis of lung disease.

Given that 77% of eligible families agreed to participate, it is likely that the EP group was representative of the entire cohort surviving during the study period. Furthermore, the incidence of BPD in our study was similar to that recently reported for the entire 2006 EPICure2 cohort^5^ emphasizing the fact that “new” BPD is very much a disease of immaturity. The ethnic mix of the EP and PTC groups was similar and reflects the local population of the multi-ethnic East London community. By using multivariable regression analysis, we were able to investigate the potential impact of ethnicity on infant pulmonary function over and above that of prematurity. There was only sufficient power to classify infants according to whether or not they were of white European descent, rather than more precise ethnic grouping, which would have required hundreds of subjects per group.^26^ Nevertheless, when we restricted analyses to those born to Black mothers (African or Afro-Caribbean origin) very similar, albeit even stronger, associations were found. Since many of the fullterm infants had been recruited to clinical research studies as controls when investigating the influence of Cystic Fibrosis on lung function, there was a much lower proportion of non-white infants in this group. However, despite the relatively small numbers, highly significant reductions in FEV_0.5_ and FVC of the same order of magnitude as seen in the preterm infants were observed, with no difference in FEV_0.5_/FVC. This suggests that the lower values seen in non-white preterm infants did not reflect “worse” lung function or lung disease, but simply ethnic differences in lung function as observed in older subjects,^26^ possibly associated with differences in body physique. Unfortunately we did not record crown-rump length in relation to crown-heel length in this study and hence are unable to comment on its potential contribution to observed differences.

Although ethnic origin has been recognized as an important determinant of pulmonary function in older subjects,^26^–^28^ with similar evidence that such differences may also occur during infancy and early childhood,^29^–^32^ the impact of ethnicity has rarely been taken into account when examining the impact of prematurity on subsequent pulmonary function. Current infant PFT reference equations, including those used in the current study,^16^,^19^ are predominantly based on white infants. Results from this study emphasize the need to expand these to other ethnic groups or to recruit appropriate controls to studies such as this.

The finding of relatively better pulmonary function in boys than girls was unexpected, as most previous studies have reported worse morbidity in male infants following preterm birth.^4^,^33^,^34^ Given that investigation of sex differences was not an a priori hypothesis and the relatively small sample size once subsets were examined according to ethnic group, gestational age sex and sex, this finding needs to be interpreted with caution, as it may simply reflect a chance finding due to sampling. In addition, these findings may be biased by the fact that a larger proportion of SGA infants were recruited to the PTC than EP group (possibly reflecting the fact that such children rarely survive if born EP) and that of these children, twice as many were girls. We and others have previously shown the adverse effects of SGA on subsequent lung function,^13^,^35^ and it is possible that while not appearing as a significant outcome on multivariable analysis, this imbalance may have contributed to the apparent and unexpected sex difference that we observed.

Given an imbalance ratio of 1.27, the sample size of 93 infants was equivalent to 45 per group,^20^ and hence adequately powered to detect differences of at least 0.5 *z*-scores between EP and PTC groups with respect to the FEFV outcomes. However, technical difficulties with obtaining acceptable FRC_pleth_ data in some infants reduced the total sample size for this outcome to 76 infants, such that a potentially important difference of 0.5 *z*-scores between groups did not reach statistical significance (Table 2). While the relatively lower FRC_pleth_ in PTC could be a chance observation, as discussed above, it could also reflect the higher proportion of SGA infants in this group.

Cancellations due to respiratory infections meant that the EP group was, on average, 10 weeks older than the PTC infants. However, use of age-adjusted reference equations, derived from healthy term infants studied over the first 2 years of life, means that this difference is unlikely to have impacted on interpretation of results. Deferment of testing for several weeks after any respiratory illness also ensured that results better reflected baseline lung and airway function rather than transient inflammation due to any recent exacerbations.

With the exception of obvious differences in birth characteristics, antenatal and postnatal care, the preterm groups were well matched for factors such as ethnic background, sex distribution and smoking exposure, all of which can potentially confound interpretation of PFTs.^9^,^13^,^32^ The incidence of maternal smoking reported in this study was lower than previously reported^32^,^35^ which may reflect recent public health campaigns and shifts in public opinion. While the very low reported incidence of smoking during pregnancy could be due to some recall bias or denial, cotinine analysis at time of test confirmed reliability of reported postnatal smoking habits. In contrast to previous reports including those from our department,^32^,^35^ there was no significant impact of maternal smoking on pulmonary function in this population, which probably relates to the relatively small numbers within each group that were exposed to passive tobacco smoke pre- or post-natally.

**Fig 4:**
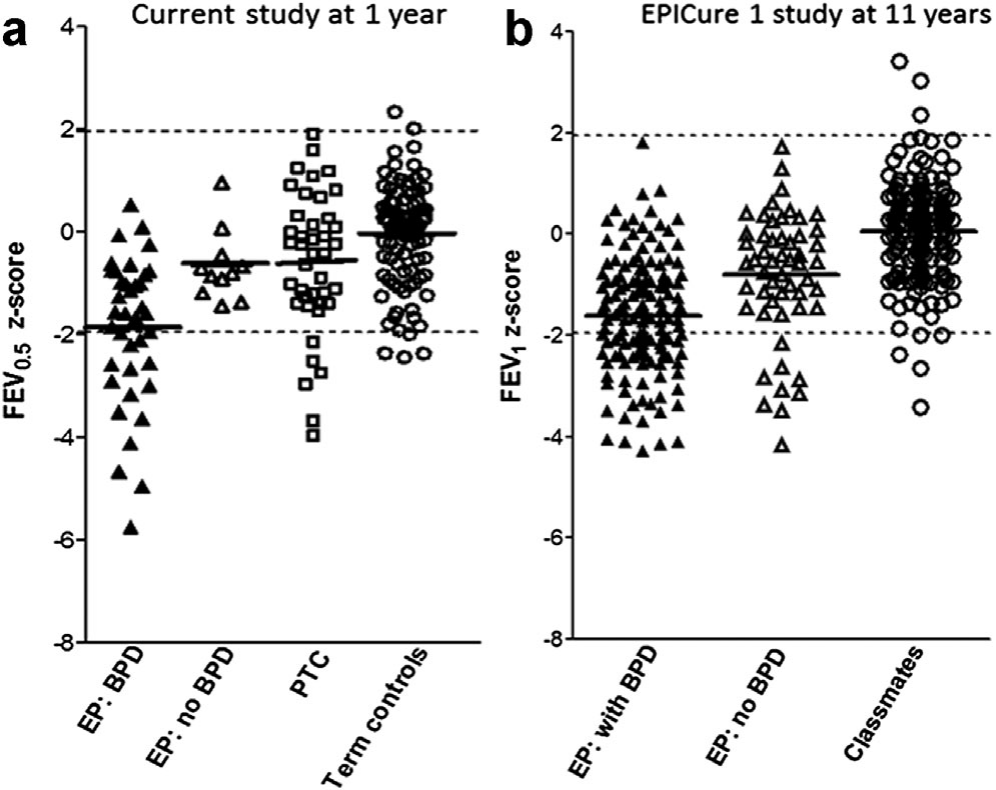
Comparison of timed forced expired volumes (FEVt) for participants in: (a) the current study at ∼1 year; (b) the EPICure 1 study^3^ at ∼11 years of age. Footnote: EP:BPD, extremely preterm subjects with bronchopulmonary dysplasia; EP:no BPD, EP subjects without BPD; PTC, preterm controls; Term controls, healthy fullterm infants; Classmates, healthy classmate controls born at term gestation. Dashed lines indicate the upper and lower limits of normality (i.e., ±1.96 *z*-scores); solid horizontal lines represent mean values for each group.

In this study, 79% of EP infants developed BPD, which corresponded to a similar percentage to that reported in both the original EPICure study^36^ and the more recent EPICure 2 study.^5^ Despite the crudeness of defining BPD simply on the basis of who remained in oxygen at 36 weeks postmenstrual age,^22^ we have previously shown this to be a remarkably sensitive means of discriminating those who go on to have long term respiratory problems.^3^,^37^ Indeed, the pattern of pulmonary function abnormalities observed in this population of infants at around 1-year of age, bears a striking resemblance to that seen at 11-years during the respiratory follow-up of the 1995 EPICure cohort^3^ (Fig. 4). It is of concern that a similar decrement of PFT values is found in the current cohort, despite improving perinatal care and survival. Since insults to the developing lung may have life-long effects, sub-optimal pulmonary function at a year of age in those born preterm may persist through to adulthood.^2^,^3^,^7^,^37^,^38^

Even in the absence of prolonged or aggressive ventilator support, the lungs of infants with “new” BPD are characterized by a simplified alveolar structure with variable degrees of fibrosis.^1^,^22^ This combination of disrupted alveolarization, contributing to poor airway tethering, and airway thickening due to fibrosis contribute to the long-term impairments of pulmonary function that have been reported after preterm birth.^1^–^3^,^37^,^39^ Similar reductions in pulmonary function have been reported in preterm infants with BPD when compared either with healthy term infants or preterm subjects without BPD,^40^–^42^ with no evidence of catch up by 2-years of age.^41^ Although recent studies have used similar equipment to measure PF in infants born preterm, with or without BPD,^42^,^43^ use of inappropriate reference equations and/or standardizing outcomes by body weight, both of which may bias interpretation of results^16^,^19^ precludes comparison with the current data.

Impaired airway function has been reported following preterm delivery even in the absence of any neonatal lung disease or ventilator support,^39^,^44^ as confirmed in this study. Such changes may persist to childhood even in relatively “late preterm” subjects,^45^ reflecting the critical developmental changes that occur in the lungs during the last trimester of pregnancy.

## CONCLUSIONS

Results from this study indicate that it is essential to account for ethnic differences in lung function when interpreting the impact of early insults to the developing lung. Despite recent improvements in maternal health and neonatal care, preterm delivery continues to have a negative impact on subsequent lung development, even in relatively “mature” PTCs. Since impairment may persist throughout childhood and into adult life, it is important that carers and the children themselves are aware of this association so that life-style is adjusted to preserve available pulmonary function. The increased risks following preterm delivery of developing more severe acute childhood respiratory illnesses, as well as early onset chronic lung disease in adulthood, also need to be considered by physicians whenever such individuals present with respiratory symptoms.
